# Malingering

**Published:** 1888-10-06

**Authors:** W. Curran


### Malingering.

By W. Curran, A.M.D.

(Continued from p. 315, Vol. IV.)

Without doubt the most important class of malingerers are those who feign mental disorders. There are none of us who would not shrink from withholding from any real malady especially a mental one, every care and attention; and when we remember how unwholesome the mental condition of the criminal classes is, and how specially prone to become diseased, we must surely study with every attention any apparent deviation from sanity, and be prepared to give the complainant the benefit of the doubt, if any exists.

The assumption of acute dementia is perhaps the most common form of deceit, and is generally easily detected. The smashing of the cell furniture and tearing up of prison clothes, the shouting and yelling until the shouter becomes tired and drops off to sleep, the plastering of the cell wall, and even the painting of the face with fæces are not necessarily signs of unsoundness of mind, and the little impression they make upon the mind of the wary doctor is evidently often a cause of considerable astonishment to the actor. There are frequently signs of preparations for the doctor's visit which at once excite suspicion. Pretended attempts at suicide are not uncommon. The preparations are generally elaborate, and are so timed as to be interrupted by the officer's visit.

A very clumsy impostor came under my own notice. An old hand who had slight chronic bronchitis, sufficient he knew to save him from corporal punishment, asked to be admitted into hospital, he having been there before during an acute exacerbation, and having evidently liked the life. I refused his request, whereupon he set to work to give as much trouble as he could; complained, sick every day, refused to do any work, and finally had me summoned to the prison in the middle of the night on a frivolous excuse. On hearing next morning that he was reported for making a frivolous complaint, he bit his arm sufficiently severely to make a slight wound, and declared it to have been done by the warder. Finding himself not believed, he refused to answer any questions, and began to slobber at the mouth and roll his eyes about in a way which he evidently thought would carry conviction to the minds of all who saw him. He had, however, made a mistake. Self-inflicted wounds are not looked upon with favour by the authorities, so he was promply removed to a padded cell and confined in a strait waistcoat. Here he first succeeded in making his nose bleed by rubbing it against the cocoa-nut matting on the floor of his cell, and then contrived to tear off the strait jacket. He then had his hands handcuffed behind his back for a week, at the end of which time he gave in, and returned to work. He behaved fairly well for the rest of his time, and although, whenever I undertook the medical officer's duties I always found him there, I never had much more trouble with him, a reminder of the handcuffs being sufficient to restore his sanity, if ever it showed signs of giving way. My last act in connexion with the prison service was attending at the local assizes to give evidence as to this man's sanity. He was being tried for felony, and when taken down to court began his old games. He declined to be tried in any other way than by court martial, and refused to cross-examine the policeman who made the charge because he had not got his side-arms on. He was sentenced to seven years' penal servitude, and offered to toss the Deputy Recorder whether he made it fourteen or nothing.

Unfortunately, all cases are not so easily detected as the one I have just related; and, still more unfortunately, cases of real insanity are occasionally mistaken for attempted imposition.

A prisoner at a certain convict prison constantly applied to the medical officer, saying that he was electrified in his inside. He was looked upon as a malingerer by an experienced prison surgeon, and reported as such to the Home Office in reply to inquiries made on his petitioning for release, on the plea that he was consumed by mental torture, etc. Some time after the prisoner was sent to Woking for further observation, and there was found to be insane.

In Marshall's "Enlisting and Discharging of Soldiers" some cases are related in which this took place. In one case a private refused to go on duty, was tried by court martial, and received 175 lashes. He was afterwards found to be insane. In another a soldier was five times court-martialed for pretending insanity, and five times received corporal punishment; he was finally discharged, and shortly after, in a fit of maniacal delirium, committed suicide by drinking sulphuric acid.

In the army it is not uncommon for recruits who repent having enlisted to pretend that they cannot learn drill. Vanity on the part of the drill sergeant will perhaps favour the assumption that anyone who does not promptly become efficient under his tuition must necessarily be weak in intellect. Whether this be so or not, the question not unfrequently arises. It is well in such cases to watch the behaviour of the soldier as a whole. If he is only untidy in dress and dirty in habits, there is no reason why rigorous discipline should not work a change for the better; but if he is weak on all subjects, unable to hold his own with his comrades, and especially if his aspect is imbecile, he had better be discharged. It is well to bear in mind, in weighing the importance of symptoms of insanity, the length of a prisoner's sentence and the amount still unexpired. Thus signs of mental aberration appearing at the beginning of a term of imprisonment will be looked upon with much more suspicion than the same symptoms arising at the end, for the obvious reason that whereas in the first case the prisoner may hope to exchange the prison for the greater freedom of the lunatic asylum, in the second no such object can be in view.

One other point too must be borne in mind. It is easy for a man to assert that he is possessed with a devil, or that his interior is a burning fiery furnace; but does he act in such a way as would a man who really imagined that he was in such an uncomfortable condition? Does he take steps to relieve himself of the devil or to quench the internal flames? If he does not suspicion should be at once aroused.

With regard to the method of dealing with cases of malingering, whether only suspected or clearly made out, not very much need be said. The only safe rule to follow is to treat the feigned ailment as a real one, but with such rigidity as to make the ordeal as trying as possible. Thus a manufactured wound should be met by strict antiphlogistic remedies, the principal one being as low a diet as is compatible with safety. If the case is not of sufficient severity to require treatment in hospital, confinement to the cell until healing has taken place will soon convince the prisoner that the game is hardly worth the candle. In cases of artificially-produced abscess, an early and free application of the knife will impress upon the patient's mind the inadvisability of further attempts in the same line of imposture. In maniacal delirium, the padded cell and strait waistcoat, or, better still, a coldshower-bath, for all prisoners are more or less hydrophobic, will bring back the actor's thoughts from flights of ethereal fancy to dull, prosaic, every-day affairs. When food is refused, the stomach-pump or the enema syringe will come into use, and if these fail the exhibition of highly-seasoned beef tea down one nostril will restore the healthy appetite in a marvellously short space of time.

Cases of wilful vomiting seldom give rise to much trouble. One such case I will relate from my own notes in conclusion. I found a boy in hospital who had been admitted for some other complaint, which had been relieved, but who could not be discharged, as he was supposed not to be able to keep down any food. Vomiting took place after each meal, and consisted entirely of undigested food. He had no abdominal tenderness, no tumour, and was not losing weight—a fact readily known in prison, where all new comers are weighed. I felt convinced that he had the power of vomiting at will, and directed that his diet should consist of half-a-pint of milk a-day, administered in tablespoonful doses. The symptoms ceased. The amount of food was grudgingly increased until the ordinary diet was returned to, when he was promptly discharged, with an intimation that a return of the symptoms would necessitate a still more prolonged and rigid application of the treatment.

Such are some of the expedients with which malingerers attempt to deceive the medical practitioner, and such are the methods by which, in my opinion, they should be met. I may perhaps say that I have not attempted to compile a professional paper, I have only sought to narrate some experiences which are not quite in the ordinary line. If I have done so, I shall be amply repaid for the slight labour which the arranging of the materials has cost.

